# Dog Owners’ Attitude toward Veterinary Antibiotic Use and Antibiotic Resistance with a Focus on Canine Diarrhea Management

**DOI:** 10.3390/ani13061061

**Published:** 2023-03-15

**Authors:** Alessia Candellone, Paola Badino, Flavia Girolami, Ugo Ala, Floriana Mina, Rosangela Odore

**Affiliations:** 1 Department of Veterinary Sciences, University of Turin, 10095 Grugliasco, Italy; 2 Nutrito Vet srl, Rosta, 10090 Turin, Italy; 3 Duregger srl, 12038 Savigliano, Italy

**Keywords:** antimicrobial resistance, dogs, canine acute diarrhea, pet owners, survey

## Abstract

**Simple Summary:**

Antimicrobial resistance (AMR) is one of the main health concerns worldwide and addressing the problem through the One Health approach is essential to manage the burden of AMR emergence and transmission. Prudent use of antibiotics and compliance to the prescribed therapy are key actions to prevent the impact of AMR in animals and humans. As regards companion animals, the effective use of antibiotics depends on the collaboration of pet-owners with prescribing vets. To improve compliance, it is important to improve pet-owner understanding of AMR-associated risks. We conducted an online survey to investigate AMR knowledge amongst dog owners with a focus on canine acute diarrhea (AD) management. AD is a good example to consider. The disease is stressful for owners that seek veterinary help for their dog’s condition. Some cases of canine AD are still treated with antibiotic courses as first-line medication despite antimicrobial administration often being unnecessary. Rather, it has been observed that dietary management and administration of nutraceuticals can positively impact on the resolution of symptoms. Respondents to our survey were reasonably aware of AMR existence and AMR-associated risks. Almost all of them agreed that treating canine AD with nutraceuticals would represent a valuable alternative to antibiotics.

**Abstract:**

An ad hoc questionnaire was designed in order to investigate AMR knowledge amongst Italian dog owners, owner expectations concerning pharmacological treatment of canine AD, and client attitudes towards and compliance with alternative strategies to antimicrobial administration. A total of 250 questionnaires were returned. Most of respondents were female, aged 36–70 and workers. More than a half of participants owned one dog with mixed breed, with Labrador retriever, golden retriever, dachshund, and border collie being the most represented breeds. On average, each dog was treated with an oral antibiotic 1.044 times per year. Intestinal diseases were among the main reasons (19%) for antibiotic prescription. Oral antibiotic courses without veterinary consultation (21%) and anticipated termination of the therapy (17.1%) were less common than reported elsewhere. The majority of respondents knew the meaning of AMR with a significant inverse association between the level of education and the tendency to administer antimicrobials without consulting a clinician (*p* = 0.004). Most of the owners expected a rapid recovery of clinical signs after a first episode of AD and accepted natural dietary supplementation for treating the condition. Ninety-five percent of the respondents believed that public funding should be spent to study AMR. Even though an acceptable degree of AMR awareness emerged, we feel that further efforts should be made to increase public AMR knowledge and to stimulate proactive measures to fight the phenomenon. On the other hand, the development of guidelines for the treatment of uncomplicated canine AD would help clinicians to rationalize antimicrobial use.

## 1. Introduction

Antimicrobial resistance (AMR) is an advancing global menace to public and animal health. Overuse or misuse of antimicrobials are established risk factors accelerating the development of AMR [1]. While in the past decades much focus has been put on the rise of resistant bacterial strains in humans and farm animals, currently there is growing attention in the veterinary community on the spread of AMR in companion animals. Pet ownership, indeed, has rapidly increased in developed countries and, nowadays, more than 60 million of cats and dogs share the household with their caregivers [2], with the latter being considered more as “family members” than as domestic animals. This change in the human–canine relationship has been accompanied by a greater attention to the nutrition of pets and a parallel evolution in the quality of veterinary care provided. In particular, the demand of a state-of-the-art medical assistance in the course of various illnesses has determined a massive use or abuse of drugs and antibiotics, contributing to the spread of antimicrobial resistance (AMR) [3,4]. 

Resistance is problematic in many pathogens and commensals of canine origin, including staphylococci, enterococci, *Escherichia coli*, and *Salmonella* [5,6,7]. For instance, in a study performed by Elnageh et al. [8], 13 out of 38 staphylococci isolated from 151 pets were identified as methicillin-resistant. As far as enterococci were concerned, strains isolated from feces collected from pets visiting the University Veterinary Hospital of Porto showed a resistance to tetracycline (67.0%), rifampicin (60.3%), azithromycin (58.4%), quinupristin/dalfopristin (54.0%), and erythromycin (53.0%) [9] and for *Escherichia coli* and *Salmonella*, percentages of single and/or multidrug AMR of 53.4% and 85.1%, respectively, have been identified from other authors [10,11]. 

The close human–animal bond mentioned above also leads to an increased risk of transmission of zoonotic bacteria and resistance determinants [2,12]. Further challenges are represented by the significant overlap with antimicrobials used in human medicine, the use of critically important antimicrobials for human health, and the off-label use of drugs in the veterinary field [13]. Despite this, the main antibiotic surveillance programs focus on food-producing animals and the risk of AMR transmission along the food chain, while only few countries have implemented ad hoc AMR-control programs in companion animals [14,15]. Accordingly, mitigation measures to reduce and prevent AMR in companion animals mainly rely on awareness by veterinarians. From this point of view, the new Regulation (EU) 2019/6 on Veterinary Medicine Products, the national prescribing guidance adopted in some countries, and the Federation of Veterinarians (FVE) and Federation of European Companion Animal Veterinary Association (FECAVA) guidelines on prudent prescription of antibiotics have reinforced the need to improve diagnosis in order to control the spread of AMR. Nevertheless, pet-owner compliance and attitudes toward antibiotic use are also important drivers to reduce AMR [16]. Vets reported that owner pressure and their expectations for a clearly defined and rapidly active treatment may favour unjustified antimicrobial prescribing. We therefore felt that, in order to identify appropriate strategies for change, it would be interesting to investigate client knowledge of antibiotics and AMR and/or their inclination to use alternatives to antimicrobials. In canine medicine, inappropriate antibiotic prescribing may concern treatment of self-limiting diseases and/or illnesses of non-bacterial origin. Amongst them, diarrhea is a common matter of distress for dog owners, who consider the cure of such condition an urgent priority. It has been estimated that approximately 7% of canine patients admitted for medical care show acute diarrhea (AD) as the major clinical sign [17,18]. Although AD tends to be self-limiting in most cases, with a mild impact on dog wellness, owners commonly ask for veterinary consultation or even resort to self-medication [18,19]. AD is most frequently related to dietary indiscretion, endoparasites, or transient uncomplicated viral or bacterial infections; however, in many patients, the exact aetiology is hard to identify and symptomatic medical management with or without dietary changes is generally adopted [20]. In this context, some cases of uncomplicated AD are still treated with a short-term antibiotic course as first-line medication, despite antimicrobial administration being shown to be often unnecessary [21,22,23]. The administration of nutraceuticals has shown promising results in treating diarrheic patients. These substances show anti-inflammatory effects and may act synergistically in the restoration of microbiota wellness while contributing to management of the symptoms [24]. Polyphenols, in particular, can be useful in modulating the gut microbial ecosystem, limiting the translocation of pathogenic bacteria and reducing the inflammation associated with colonic mucosal damage [18]. However, standardized protocols and/or official guidelines for proper management of canine AD are quite inconsistent. Given the above scenario, this survey aimed to investigate: (1) AMR knowledge amongst dog owners; (2) owner expectations concerning pharmacological treatment of canine AD; and (3) client attitudes towards and compliance with alternative strategies to antimicrobial administration. 

## 2. Materials and Methods 

### 2.1. Participant Recruitment and Survey Design

An online cross-sectional survey was administered from April 2020 to November 2020 to dog owners living in Italy, to investigate their perceptions of the AMR emerging issue and of antimicrobial use, especially with respect to canine AD. 

The questionnaire was created in the Italian language using Google Forms©, (accessed on 10 March 2019), (see Survey, File S1) and it was shared on social media (Facebook©, accessed on 30 April 2020 ) and distributed via email and instant messaging services (WhatsApp© and Messenger©). The survey was organized into three separate sections: (1) demographic and epidemiologic section (14 questions); (2) antimicrobial usage section (16 questions), and (3) AMR perception section (5 questions). 

In the first section an initial set of demographic questions was asked. These included gender, age, residential code, level of education, employment status, and family status. Only volunteers aged more than 18 years were considered eligible for enrolment. Respondents were arbitrarily categorized into two classes: 18–35 years and 36–70 years. With respect to employment status, respondents were classified as “workers” (i.e., part-time or full-time professionals), “non-workers” (i.e., full-time students or unemployed people), or “retired/homemakers” (see Survey, File S1).

Owners were then asked about the dog or dogs owned (number and breed of each), species of animals kept other than dogs, and caregiver attitude for referring their dogs to a trusted veterinarian or a specialist (see Survey, Section 1, File S1). 

In the second section, online respondents were asked about their habits of antimicrobial administration per os and their knowledge about when and how a canine disease could benefit from an antibiotic treatment. Caregiver perception of antimicrobial utility and risks in the course of various illnesses affecting canine patients, with particular emphasis on AD, were also investigated (see Survey, Section 2, File S1). 

The last section focused on owner awareness of AMR. In detail, respondents were first asked if they knew the meaning of AMR; a definition of AMR was then provided and further questions about associated risks were asked (see Survey, Section 3, File S1).

No ethics approval within either national or EU legal systems was needed for this study as enrolment was on a voluntary basis and the participants consented to anonymous information collection as per General Data Protection Regulation (Regulation (EU) 2018/679). Respondents agreed to participate in the study voluntarily by self-enrolling. They were informed that their answers would be published in a scientific paper. By completing and returning the survey, they agreed to the inclusion of their data.

### 2.2. Data Analysis

The data collected in the survey were transferred to a spreadsheet (Excel, Microsoft) and underwent descriptive analysis. Results are reported as frequency (n/N) and percentage (%). Some items were further analyzed to assess a relevant association. In detail, the considered variables were: (a) the influence of the educational level on AMR awareness, (b) the influence of the educational level on AMR concern, (c) the influence of the respondent age on AMR concern, (d) the influence of education level/age on the administration of oral antimicrobials without consulting a veterinarian, (e) the influence of education level/age on the number of antimicrobial courses stopped before the end of the treatment, and (f) the influence of the employment status on the respondents’ willingness to personally fund academic research into AMR. The statistical analysis, based on either the chi-square test or Fisher’s exact test for small samples, was carried out by using “R”, a free software environment for statistical computing and graphics (version 3.6.3), and ORs with 95 percent confidence interval are reported for significant results. Statistical significance was set at *p* < 0.05.

## 3. Results 

### 3.1. Demographics

A total of 250 completed questionnaires was returned. The demographics of respondents are summarized in Table 1. Most of respondents were female (79%) and aged 36–70 (60%). Almost all the respondents were Italian (96%). According to the answers, the level of highest education was classified as “University”, including those with a bachelor’s or a master’s degree, or “Non-University”, related to a middle- or a high-school certificate. The two classes were homogeneously distributed with a slight prevalence of “Non-University” respondents (51%). The employment-status categories were concentrated into three main classes: “workers”, including full-time, part-time, and occasional workers, “unemployed”, including students and unemployed people, and “retired/homemakers”. The “workers” category represented the majority of the participants (77%). More than half of the participants owned only one dog (59.6%), 26.3% of respondents stated that they owned two dogs, whereas 14.2% stated that they took care of three or more dogs. The most represented breeds were mixed breed (34%), Labrador retriever (4.6%), golden retriever (4.6%), dachshund (3.6%), and border collie (3.1%). Moreover, 50% of the survey respondents (125/250) were also responsible for the care of species other than dog. For instance, 78.3% (97/125) kept a cat; 12.5% (16/125) kept a fish; and around 10% (12/125) kept a turtle. Subscribing to pet insurance was quite uncommon. Of the respondents, 29% declared that they were the sole caregiver of his/her dog(s). The great majority (96%) had a trusted veterinarian, and 49% had also referred their dog(s) to a specialist veterinarian. On average, the respondents took their dog(s) for a veterinary consultation three times per year (mean = 2.9 times).

### 3.2. Antimicrobial Use

**Figure 1:**
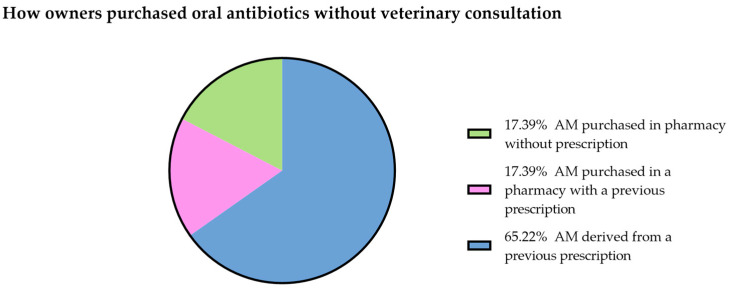
How owners purchased oral antibiotics without veterinary consultation.

**Figure 2:**
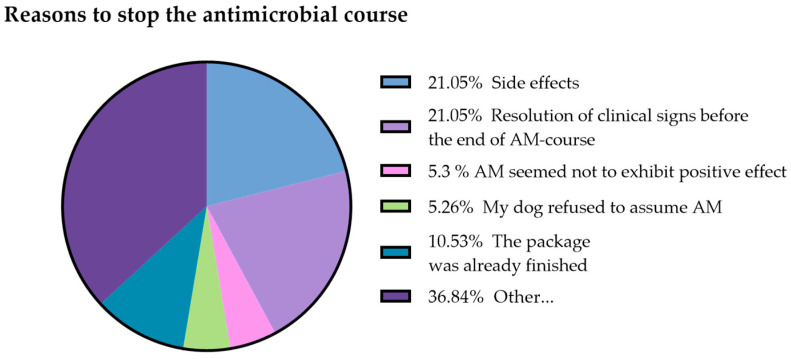
Reasons for stopping antibiotic administration before the end of treatment.

Forty-four percent (111/250) of respondents had administered at least one oral antimicrobial course over the previous 12 months. On average, each dog was treated with an oral antibiotic 1.044 times per year and 23 respondents out of 111 (21%) admitted to having administered an oral antibiotic course without a veterinary consultation. Among them, 65% (15/23) administered drug leftovers from previous prescriptions, 17% (4/23) purchased the antibiotics at the pharmacy without prescription, and 17% (4/23) purchased the antibiotics using an old prescription (Figure 1). According to the respondent answers, the veterinarian prescribed an oral antimicrobial course because of post-surgical prophylaxis 20% (18/88), intestinal disease 19% (17/88), skin disease 13.6% (12/88), oral cavity pathology 9% (8/88), parasitic disease 9% (8/88), urinary infection 9% (8/88), miscellanea 7% (6/88), respiratory disease 6% (5/88), ear infection 4% (3/88), genital disease 1% (1/88), ab ingestis pneumonia 1% (1/88), and allergic disease 1% (1/88), Table 2. Nineteen (17.1%) respondents out of 111 declared having stopped antibiotic administration before the treatment protocol was completed. The main reasons for termination of the therapy were: presence of side effects (21.1%, 4/19), had finished the pack before the end of the treatment (10.15%, 2/19), the symptoms had already disappeared (21.1%, 4/19), antibiotic seemed to not be working (5.3%, 1/19), and other non-specified reasons (36.8%, 7/19). In one case the treatment was discontinued because the dog refused to take the drug (Figure 2). Ten percent of the total number of respondents (25/250) would take their pet to another veterinarian if their veterinarian refused to prescribe an antibiotic treatment.

Two values in the figure (“AM was given by a family member or a friend” and “AM was purchased on internet”) were not plotted because they were zero. AM: antimicrobials.

### 3.3. AM: Antimicrobials

**Figure 3:**
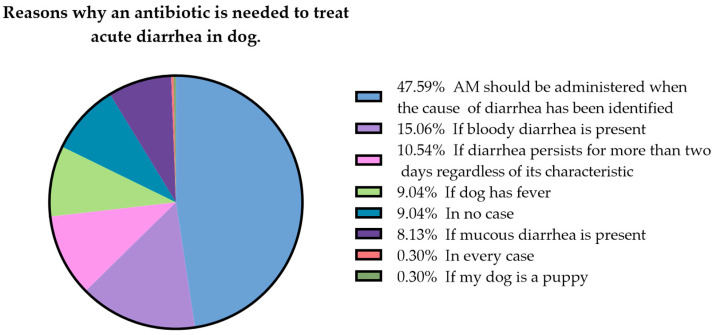
Reasons why, according to respondents, an antibiotic is needed to treat acute diarrhea in dogs.

**Figure 4:**
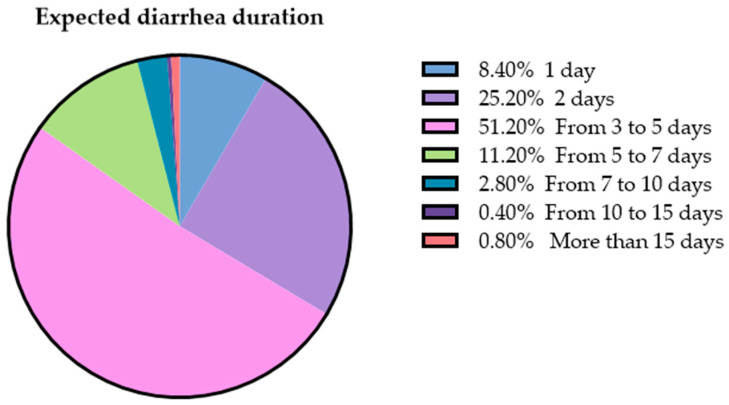
Expected acute diarrhea duration according to respondents.

Respondents were also asked to what extent an oral antibiotic is needed to treat dog AD; multiple answers were possible. Forty-seven percent (158/250) said that antimicrobial treatment should be administered when the cause of diarrhea has been identified; 15% (50/250) thought that it is recommended in the case of bloody diarrhea; 10% (35/250) said that it is needed if diarrhea persists for more than two days regardless of its characteristics; 9% (30/250) thought that it is needed if the dog has fever; 9% (30/250) said that it should not be used; and 8% (27/250) said that it should be used in cases of diarrhea with mucus (Figure 3). Most owners expected a rapid recovery of clinical signs after the first episode of AD, with 51% seeking resolution of clinical signs within 5 days (Figure 4). Thirteen percent of dog owners would still give antibiotics to their dog/dogs to treat diarrhea even knowing that these molecules are not free from side effects and 30% would still administer the antimicrobial therapy even knowing that it may represent a risk for human health. Knowing that natural dietary supplements can help the resolution of diarrhea without any side-effects, 92% of respondents said that they would accept this treatment approach, while only 2% (4/250) would not.

A value in the figure (“If my dog was senior”) was not plotted because it was zero. AM: antimicrobials.

### 3.4. Antimicrobial-Resistance Perception

Eighty-four percent of respondents knew the meaning of AMR and the large majority (96%) thought it was a major problem. Owners felt that public research is needed to face the AMR challenges (97%). Ninety-five percent believed that public funding should be spent in this field but only 33% of them would have been willing to personally fund targeted public research. 

No significant relationship (*p* = 0.2) was found between knowledge of what AMR means and the level of education or between the education level and the belief that AMR is a problem of major concern (*p* = 1). No significant relationship emerged between the age of respondents and the belief that AMR is a major problem (*p* = 0.99). Otherwise, the education level significantly inversely influenced the attitude to administer an oral antimicrobial without consulting a veterinarian (*p* = 0.004 and OR = 0.24, confidence interval 0.07–0.70) and a trend was shown with respect to age (*p* = 0.07). No significant relationship was observed between the age of respondents (*p* = 0.27) and the number of antimicrobial courses stopped before the end of the treatment. There was also no relationship between employment status and respondents’ willingness to personally fund academic research into AMR (*p* = 0.82). However, among the “worker” category, 37% (72/193) said that they would personally fund the research, 48% (93/193) answered ‘I don’t know’, and 15% (28/193) would not be willing to fund it. Among the “unemployed” class, 22% (7/32) answered positively, 66% (21/32) did not know, and 12% (4/32) answered negatively. Among the “retired/homemaker“ class, 20% (5/25) answered they would fund academic research, 20% (5/25) they would not, and 60% (15/25) were doubtful.

## 4. Discussion

In recent years, there has been an increasing interest in the study of AMR in pets and many efforts have been made to identify AMR determinants and predictors [15,16]. While appropriate antibiotic prescription practices and the training of the healthcare professionals have been recognized as essential measures to reduce AMR, it has been postulated that the use of antimicrobials can be also affected by the interaction of knowledge of users and prescribers as well as by the compliance with antibiotic treatment among the public [25,26]. As a result, different authors have recently investigated pet-owner knowledge and expectations concerning antimicrobial use and AMR [27,28,29,30]. Our research fits into this field of study providing original data from a southern European perspective. In Italy both the sale of antibiotics and the prevalence of resistant bacteria are higher than in other European regions, and different strategies have been implemented over recent years to tackle AMR in the human and veterinary sectors [31]. A crucial aspect of AMR stewardship is access to antibiotics and the need to improve awareness and understanding on AMR among citizens, with a special focus on the One Health dimension [32]. 

Different aspects make dog owners an interesting population sample for the purposes of this study. In Italy, dogs are among the predominant pets [33]. Moreover, according to Joosten et al. (2020) [32] dogs receive antibiotic treatment more frequently than cats with an odds ratio for dogs of 2.2. 

Our descriptive analysis showed that most survey respondents were female and about one third were the primary caregiver of their dog. This result is consistent with earlier observations and with the ownership profiles of the Italian dog population [32,33,34,35]. As already observed in other countries, it seems that women are the predominant pet-care decision-maker in most households [25]. Additionally, the overrepresentation of female respondents supports the observation that women are more likely to participate in mail surveys than men [36]. 

In our study 60% of participants were aged 36–70 years. The data are partially in agreement with some previous reports and may reasonably reflect the demographic evolution of the Italian population. In the study by Vinassa et al. (2020) [35] about 70% of the respondent Italian pet owners were over 35 years of age. The educational level and the employment status of the respondents were in line with the study of Slater et al. (2008) [33] with a clear prevalence of workers. 

The majority of respondents owned one dog, belonging mainly to a mixed breed, and half of these dogs cohabited with at least another animal species. These data are in line with Carvelli et al. (2020) [30] who reported that in Central Italy almost half of the interviewed people (47%) owned at least one dog, male, crossbreed which attended a veterinary visit one to two times per year. Our study, however, showed that, on average, Italian pet owners take their dog/dogs to veterinary consultations three times per year, a higher number compared to data reported in surveys carried out elsewhere. According to an online survey conducted in 2019 in New Zealand among pet owners the mean veterinary consultations over 12 months was 1.67 times [36]. However, the 2017–2018 U.S Pet Ownership & Demographics Sourcebook reveals that in the United States mean dog veterinary visits per household per year is 2.4. Bir et al. (2020) [37] reported that 35% of owners seek veterinary care more than once a year, while according to Dotson and Hyatt (2008) [38] the average dog visits the veterinarian twice as often as does the average cat. Moreover, as the probability of visiting a veterinarian increases with age and income of dog owners, our finding seems to be consistent with the demographic data of participants. The data are also in line with the observations by Slater et al. (2008) [32]. By studying pet ownership and pet management patterns in central Italy they found that the median number of visits for dogs was one to three. It seems that the Italian dog owner’s attitude toward pet care has not significantly changed over time. Finally, it should also be considered that about 60% of the respondents to our survey said that they owned one dog. This could impact on the availability of time and resources to bring the dog to a veterinary consultation in an emergency as well as for preventive purposes.

Our survey demonstrated that each dog had been treated with an oral antimicrobial course 1.044 times over the previous 12 months and 17.1% of participants shortened the recommended duration of antimicrobial treatment. A study conducted in Portugal between 2018 and 2019 showed that only 60% of pet owners completed the full course of treatment recommended by the veterinarian despite administering antimicrobials at the prescribed dose [39]. Despite our data depicting a more reassuring situation than that of Portugal, they still underline how compliance with treatment remains a concern in both human and veterinary medicine. In a recent survey of the population knowledge and attitude toward human antibiotic usage in western Saudi Arabia it was noted that only 38.2% of respondents realized the need to complete the course of treatment, and a percentage between 30% and 72% of participants did not complete the antimicrobial course. The main reasons for not completing the therapeutic protocol were respondents feeling better and thinking that the used antibiotics did not work [40]. In veterinary medicine a variety of factors can influence the observance of the therapeutic recommendations. Some practical barriers, such as odd tablet size, difficulty in the administration, timing of dosing, and cost play an important role in the shortening of an antimicrobial course [30]. Concern about overmedicating the pet also represents a reason for not following vet directions [28]. On the other hand, it has been proven that discussing the dosing regime in light of the owner’s circumstances and providing them with an information-sheet about appropriate antibiotic therapy are simple and inexpensive strategies to increase the rate of compliance [41].

Although in Italy antibiotics must be sold exclusively with a prescription by a health professional, according to our findings 21% of participants still resort to antimicrobial administration without consulting a veterinarian. This lower percentage compared to other countries, could reflect a good knowledge of the importance of antibiotic prescription even though it cannot be excluded that respondents were less likely to admit self-medication practices. The majority of Greek small-animal veterinarians participating to a survey declared that pet owners frequently administer antibiotics before bringing the animal to the practice [42]. The main reasons were consistent with those in this survey. Drugs administered are mainly leftovers from previous treatments, purchased at the pharmacy with a previous prescription, or purchased without any prescription. The rapid expansion of the internet in the last few years has also increased the possibility of purchasing antibiotics for veterinary use online. It has been observed that more than a half of websites do not require a valid prescription and the quantity of drugs is not limited to a certain number of packages [15]. The availability of leftover antibiotics often results from either patient/pet non-compliance or dispensing of a larger number of tablets than needed for a single course [43]. According to the study of Redding et al. (2019) [28], 13% of interviewed dog owners admitted keeping unused antimicrobials on hand and administering them in the case of a flare-up of a chronic condition (e.g., otitis or dermatitis). However, administering remnants for a later infection in the same patient carries a double risk: the subsequent pathogen may not share the susceptibility of the original one and the medication may have lost potency due to improper or prolonged storage [44]. 

Ten per cent of respondents to our survey declared they would take their pet to another veterinarian should the consulted professional refuse to prescribe an antibiotic treatment. This is not surprising as it has already been reported that both physicians and veterinarians often perceive patient/owner demand for antibiotics [45,46]. A study of 25 US pet-owners found that, in a scenario where antimicrobials may not be effective, most (*n* = 21) would still like them to be prescribed [28]. Owner expectations of antibiotics may reflect the lack of understanding of the risks associated with antimicrobial drugs as well as the self-centered attitude that their pet should receive medication independently of general AMR concerns [31]. More effective vet–owner communication strategies may serve to prevent pressure and mitigate the problem of sub-optimal prescriptions. 

We felt intriguing to investigate owner perception about antimicrobial administration in cases of dog AD as the symptom represents one of the most common causes of veterinary consultation in dogs in Western countries. Moreover, even though microbiological or parasitological tests are rarely carried during such emergencies, use of systemic antimicrobials is quite common. According to Singleton et al. (2019) [17] systemic antimicrobials are prescribed in 49.7% of cases of dog AD. In our study, intestinal diseases account for 20% of antibiotic treatments. However, it was not possible to ascertain if they were acute conditions. Our results show that a considerable percentage of respondents consider diarrhea persisting for more than two days, bloody diarrhea, and/or diarrhea with mucus, as conditions requiring an antibiotic course. Moreover, 27% of participants expect complete recovery from AD within 48 h after the first episode. In contrast to owner-belief, the normal duration of AD can range from 5 to 7 days and the condition likely resolves without any medical intervention [20]. Rather, provision of dietary modification advice and gastrointestinal nutraceuticals alone are positively associated with resolution [17,18]. As an example, probiotic administration reduced the duration of symptoms compared with placebo in acute self-limiting cases of canine diarrhea [47]. It has been recently demonstrated that canine AD is associated with redox imbalance and the dietary administration of antioxidants could support the management of the condition and prevent the onset of chronic gastrointestinal diseases, thus reducing the use of antibiotics [18]. As our results suggest, this latter approach would also intercept with the public growing interest in the use of natural compounds to bring health benefits in both the prevention and the treatment of disease. Taking into account the aforementioned and the availability of alternative treatment options to antimicrobials, it would be desirable to achieve globally accepted guidelines for canine AD management and to improve the knowledge of untrained practitioners to reducing inappropriate interventions and the risk of AMR. 

The present study suggests the existence of a not negligible level of public awareness and concern about AMR. A high percentage of dog owners feel that AMR is a major problem. Nevertheless, 16% of respondents admitted they did not know about AMR, and a small percentage of respondents seemed to be less worried about the impact of AMR on themselves or their families than on their pet’s health. Our findings agree with previous reports describing improvement in Italian pet-owner knowledge related to AMR, while also stressing the need to further develop awareness and responsibility at an individual level [48]. It has been described elsewhere that few pet owners have an understanding that AMR is an issue for animals and a very small minority know about interspecies transmission [27]. From a veterinary perspective, it could be helpful to raise the awareness of AMR as a One Health problem and to provide appropriate information about the risk of sharing resistant bacteria between pets and humans. In particular, veterinarians should inform pet owners that AMR can be hindered by properly administering their pets with antimicrobials, by completing the prescribed treatment, and by using these drugs only for proven bacterial infections and after medical prescription. In addition, it will be crucial to make the public conscious that even one person can support the fight against AMR, as, for instance, participating in surveys aimed at collecting epidemiological and social data, such as the one described in the present paper. 

The significant relationship between prudent administration of antimicrobials and level of education was expected. A cross-sectional survey among Italian adults had in fact already demonstrated that higher education levels are predictors of good knowledge about antibiotics and AMR [49]. 

Our survey provided evidence that most respondents thought that public research is needed to deal with the AMR problem and 95% believed that public funding should be spent in this field. By contrast, only one third (33%) of the respondents were willing to personally fund the research. As this survey was carried out in the midst of the COVID-19 crisis, the results may be partially explained by the emotional states that frequently mark pandemics, such as uncertainty and economic anxiety. On the other hand, citizen engagement in AMR research should be further stimulated at different levels. Within most national action plans for AMR, citizens are expected to be recipients of awareness activities or education interventions rather than actively engage in proactive self-care measures to reduce the need of antibiotics [50]. In some countries the societal mission of universities has been long neglected and the broadening of science-communication activities by academics should be strengthened. This could, in turn, motivate citizens to participate to AMR scientific projects and even to donate to AMR crowdfunding.

### Limitations

For ethical reasons our research relied on voluntary participation. This could have implied engagement by highly committed owners with a higher-than-average perception on risks associated with antimicrobial usage and AMR. One must also consider that although the definition of AMR was provided in the questionnaire, its thorough understanding can be challenging. In addition, social desirability bias cannot be excluded. 

## 5. Conclusions

Our data provide information concerning dog owners’ perceptions of AMR-associated risks and challenges. Compared with previous studies carried out elsewhere, an acceptable degree of awareness emerged. Nevertheless, further efforts should be made to increase public AMR knowledge and concern and to stimulate proactive measures to fight the phenomenon. This, in turn, would positively affect the individual propensity to fund public research on the topic. The focus on canine AD allowed us to investigate pet-owner willingness to use natural dietary supplements instead of antimicrobials to manage this clinical condition. Both in humans and in veterinary medicine, probiotics, prebiotics, postbiotics, and antioxidants are showing promising results in the treatment of gastrointestinal diseases. The outcomes of our survey are rather encouraging as the majority of respondents demonstrated a high propensity to use this alternative approach. We suggest that the development of globally accepted guidelines for the treatment of canine AD would help clinicians to rationalize antimicrobial use. Likewise, vets should be aware of their role in educating owners to compliant antibiotic use and in improving caregivers’ engagement in the AMR fight, by promoting, for instance, participation in surveys distributed by medical facilities or research institutes. 

A possible limitation of the study could be the method of enrolling respondents, as only owners who voluntarily filled in the questionnaire were recruited. Moreover, the online recruiting process could also have selected only that part of the Italian population that is more active on social-media platforms, possibly excluding caregivers with poor informatics skills.

## Figures and Tables

**Table 1:** Demographics of respondents.

Demographic Data	Categories	Percentage
**Gender**	Female	79%
	Male	21%
**Age group**	18–35 years	40%
	36–70 years	60%
**Nationality**	Italian	96%
	Other	4%
**Highest education**	University	49%
	Non-University	51%
**Employment status**	Workers	77%
	Unemployed	13%
	Retired/homemakers	10%
**Pet insurance**	Yes	29%
	No	71%
**Dogs owned**	One	59.6%
	Two	26%
	Three	7.6%
	Four	4.4%
	Five or more	2.4%
**Pets owned**	Only dog(s)	50%
	Dog(s) + others	50%
Number of veterinary consultations/year	0	1%
	1	18%
	2	28%
	3	20%
	4	8%
	5 or more	25%

**Table 2:** Reasons why oral antimicrobials have been prescribed by the veterinarians. Data are expressed as percentage of the total number of dogs who had received antimicrobials in the previous 12 months, as declared by respondents.

Reasons Why Antimicrobials Had Been Prescribed	Percentage (%)
Post-surgical prophylaxis	20
Intestinal disease	19
Skin disease	13.6
Oral-cavity pathology	9
Parasitic disease	9
Urinary infection	9
Miscellanea	7
Respiratory disease	6
Ear infection	4
Genital disease	1
Ab ingestis pneumonia	1
Allergic disease	1

## Data Availability

The data presented in this study are available on request from the corresponding author. The data are not publicly available due to privacy reasons.

## Supplementary Materials

The following supporting information can be downloaded at: https://www.mdpi.com/article/10.3390/ani13061061/s1, File S1: original survey (English translation).
