# Supplementary material for: Dog Owners’ Attitude toward Veterinary Antibiotic Use and Antibiotic Resistance with a Focus on Canine Diarrhea Management
Source: Animals (Basel). 2023 Mar 15;13(6):1061. doi: 10.3390/ani13061061 (PMC10044205; doi:10.3390/ani13061061)
Supplement: Supplementary file 1 [file animals-13-01061-s001.zip › animals-2249608-supplementary.pdf]

RESEARCH PROJECT "GO FAR!"

(Fighting Antimicrobial Resistance in dogs)

DEPT. OF VETERINARY SCIENCES, UNIVERSITY OF TURIN, ITALY

*mandatory field

SECTION 1: DEMOGRAPHIC AND EPIDEMIOLOGIC SECTION

1. How old are you? [Two digits input for age] * $

____

2. What is your gender? *

- Female
- Male
- Not declared

3. What is your Nationality [Please digit] *

____

4. If Italian, please enter your residential code [Please digit]

5. What is your highest level of schooling/education? *

- Primary school§
- Middle school§
- High school or equivalent§
- University Degree&
- Post-graduate University Degree (e.g., Masters, Doctorate, PhD)&

6. How many dogs do you have? *

- 1
- 2
- 3
- More than 3

7. Please, specify breed, sex and age of each dog owned, identifying them as Dog1, Dog2, Dog3, * etc...

8. Do you own pets other than dog/dogs? *

- Yes
- No

9. Please specify species of pets owned other than dog [select all that applies]

- cat
- horse
- fish
- turtle
- bird
- rabbit
- rodent
- other

10. Did you subscribe a pet insurance? *

- Yes
- No

11. Are you the sole caregiver for your pet? *

- Yes
- No

12. What is your employment status? *£

- Working full time
- Working part-time
- Working on a casual basis
- Unemployed
- Home duties
- Student
- Retired
- Permanently unable to work due to disabilites

13. Do you have children? *

- Yes
- No
- Not yet, but I would love to

14. Do you have a trusted veterinarian (Vet) providing care to your pet? *

- Yes, I have a trusted Vet
- No, I often change Vet

15. How often do you ask for a veterinarian consultation over a 12-month period? *

- 0
- 1
- 2
- 3
- 4
- More than 4

16. Do you have a Board Certificated Vet ("Specialist") providing care to your dog? (i.e. Nutritionist, Ophthamologist, etc...). *

- Yes
- No

SECTION 2. ANTIMICROBIALS SECTION

The next few questions are about giving your dog ORAL antibiotics (such as amoxicillin, penicillin, metronidazole, etc…). Oral antibiotics are taken by mouth, such as tablets, capsules, and syrups. This does NOT include antibiotic creams, ointments or eye drops.

17. In the past 12 months how many times has/have your dog/dogs been treated with a course of ORAL antibiotics? Please answer separately for each dog. Please enter zero for none [Open field. Identify each dog as Dog1, Dog2, Dog3, etc..]

18. As concerns the above mentioned treatments (oral antibiotics during the last 12 months), * how many of them were prescribed by a veterinarian? Please enter zero for none [Open field]

19. For what reasons did the veterinarian prescribe ORAL antibiotics to your dog/dogs? * Please briefly describe motivations [Open field]

20. How many ORAL antibiotic courses were stopped BEFORE the treatment protocol was * completed? Please answer separately for each dog, identifying them as Dog1, Dog2, etc....

Please enter zero for none. [Open field]

21. How many antibiotic courses, if any, were administered BEFORE OR WITHOUT consulting a veterinarian? Please enter zero for none. [Open field]

22. Where did you get the oral antibiotics to be administered BEFORE OR WITHOUT consulting a veterinarian? Please select all that applies.

- [ ] The antibiotics were left-over from a previous prescription
- [ ] The antibiotics were given to me by a family member or friend
- [ ] The antibiotics were purchased from a pharmacy with a PREVIOUS prescription
- [ ] The antibiotics were purchased from a pharmacy WITHOUT a prescription
- [ ] The antibiotics were purchased online without a prescription

23. For what reason/s did you stop giving the antibiotics? Please select all that apply.

Seleziona tutte le voci applicabili.

- [ ] My dog was having side effects from the antibiotics
- [ ] My dog's symptoms had already disappeared
- [ ] The antibiotics didn't seem to be working
- [ ] My dog refused to take the antibiotics
- [ ] The antibiotics pack was already over before completing the course of antibiotics
- [ ] Other…

24. To what extent do you think oral antibiotics are needed to treat the following conditions in dogs? Please select all that apply

- [ ] Viral infections, in general
- [ ] Bacterial infections, in general
- [ ] Cough
- [ ] Sneezing
- [ ] Nasal discharge
- [ ] Vomiting
- [ ] Diarrhea
- [ ] Wounds
- [ ] Otitis
- [ ] Fever
- [ ] Other

24. To what extent do you think oral antibiotics are needed to treat DIARRHEA in dogs? *
Please select all that apply

- [ ] Diarrhea with mucus
- [ ] Bloody diarrhea
- [ ] If diarrhea persists for more than 2 days, independently from its characteristics
- [ ] Always
- [ ] Never
- [ ] When the cause of diarrhea has been already identified
- [ ] If my dog is a puppy
- [ ] If my dog is senior
- [ ] If my dog has fever

25. As regards DIAR RHEA, how soon after the first episode do you expect the complete resolution?

- 1 day
- 2 days
- from 3 to 5 days
- from 5 to 7 days
- from 7 to 10 days
- from 10 to 15 days
- More then 15 days

26. Antibiotics could help the resolution of DIARRHEA, nevertheless they are not free from side effects. Knowing this, would you still give antibiotics to your dog for treating diarrhea? *

- Yes
- No
- I don't know

27. Antibiotics could help the resolution of DIARRHEA, but they may represent a risk for human health. Knowing this, would you still give antibiotics to your dog for treating diarrhea? *

- Yes
- No
- I don't know

28. Natural dietary supplements could help the resolution of DIARRHEA, without side effects. Knowing that, would you give a dietary supplement INSTEAD of antibiotics to your dog for treating diarrhea? *

- Yes
- No
- I don't know

29. Believing that your dog needs antibiotic treatment and in the case your Vet does not prescribe it, would you take your dog to another Veterinarian? *

- Yes, I would take my dog to another Vet
- No, I would trust my Vet

30. Do you think, antibimicrobial- resistance is a major problem worldwide? *

- Yes
- No
- I don't know

31. Have you heard of the term 'antibiotic resistance' *

- Yes
- No

<u>SECTION 3. ANTIMICROBIAL RESISTANCE SECTION</u>

Antimicrobial- resistance is the ability of bacteria to resist the effects of antibiotics – that is, the germs are not killed, and their growth is not stopped. Infections with antimicrobic resistant bacteria are difficult to treat.

32. Do you think Academic Research is needed to face the antimicrobial-resistance problem? *

- Yes
- No
- I don't know

33. Do you think public funding need to be spent to face the antimicrobial-resistance problem? *

- Yes
- No
- I don't know

34. Would you be willing to personally fund Academic Researches to face antimicrobic-resistance problem? *

- Yes
- No
- I don't know

35. Are you worried about the possible impact of antimicrobial-resistance on your health and your family’s one? *

- Yes
- No
- I don't know

36. Are you worried about the impact of antimicrobial-resistance on your dog/pet health ? *

- Yes
- No
- I don't know

I AUTHORIZE THE DEPARTMENT OF VETERINARY SCIENCES, UNIVERSITYOF TURIN, TO USE INFORMATION PROVIDED BY FILLING THIS SURVEY FOR SCIENTIFIC AND RESEARCH PURPOSES *

- Yes
- No

LEGEND:

& during analysis grouped in age classes ('18-35 years', '36-70 years')
§ during analysis merged as "Non university degree"
& during analysis merged as "University degree"
£ during analysis grouped in working employment status ('working', 'non-working', 'retired-home working')
